# Metabolic regulation of interferon-mediated innate antiviral immunity

**DOI:** 10.3389/fimmu.2025.1680688

**Published:** 2025-10-08

**Authors:** Tian Zhong, Qi Cao, Zhiyue Ma, Caiyu Jiang

**Affiliations:** ^1^ Department of Pulmonary and Critical Care Medicine, Sichuan Provincial People’s Hospital, University of Electronic Science and Technology of China, Chengdu, China; ^2^ Department of Assisted Reproductive Medicine, Sichuan Provincial People’s Hospital, University of Electronic Science and Technology of China, Chengdu, China; ^3^ Otolaryngology Department, Sichuan Provincial People’s Hospital, School of Medicine, University of Electronic Science and Technology of China, Chengdu, China; ^4^ Department of Respiratory and Critical Care Medicine, Sichuan Provincial People’s Hospital, School of Medicine, University of Electronic Science and Technology of China, Chengdu, China

**Keywords:** viral infection, type I interferons, type III interferons, innate immunity, metabolites

## Abstract

Respiratory viral infections pose a major global public health challenge, with pathogens such as influenza viruses, novel coronaviruses, and respiratory syncytial virus exerting serious threats to human health. These infections often progress to severe disease in children, the elderly, and individuals with compromised immunity. Due to their anatomical exposure and relatively weak mucosal defenses, the upper respiratory tract—including the nasal cavity and throat—serves as a primary portal of entry for these pathogens. Such infections can lead to otolaryngological conditions such as anosmia and otitis media, and may further exacerbate illness in susceptible populations. After viral invasion, the host perceives pathogens through pattern recognition receptors (PRRs), rapidly activating the expression and signaling pathways of type I and type III interferons (IFN). This plays a core role in antiviral defense. Notably, viral infection profoundly reshapes the host cell’s metabolic network, involving widespread alterations in carbohydrate, lipid, amino acid, and nucleotide metabolism. During this process, key metabolic products are released or generated. These are the products of metabolic reprogramming and important immune regulatory molecules that can directly or indirectly modulate the host’s antiviral immune response, particularly the interferon pathway. These findings reveal that viral invasion, host metabolic reprogramming, and interferon-mediated antiviral immunity form a tightly intertwined, and dynamically interact a regulatory network of”virus-metabolism-IFN”. This profoundly elucidates the underlying regulatory logic of the metabolic microenvironment in antiviral immunity. Understanding this mechanism offers new perspectives for diagnosis and treatment: targeting metabolic nodes or utilizing metabolic modulators, as well as combined strategies with IFN, and may become novel approaches for the prevention and treatment of upper respiratory viral diseases. This review focuses on the core role of IFN-mediated innate immunity in viral defense and its interactive regulation with metabolic reprogramming. It reviews the progress of studies on how metabolic products regulate the IFN pathway and antiviral responses through various mechanisms, and explores the potential clinical application prospects of metabolic regulation in local immune defense and the prevention and treatment of viral infections.

## 1 Introduction

Respiratory viruses represent a major global public health challenge and constitute a key component of the overall disease burden (1). Influenza viruses, rhinoviruses, respiratory syncytial viruses, and novel coronaviruses are widely prevalent worldwide, and the upper respiratory tract—owing to its anatomical exposure and relatively fragile mucosal barriers—frequently serves as the primary portal of entry for these pathogens (2–4). In the otolaryngological region, the pathogenic effects of viruses exhibit significant tissue specificity and pathological diversity. For example, severe acute respiratory syndrome coronavirus 2 (SARS-CoV-2) specifically targets olfactory and gustatory-related tissues, leading to symptoms such as anosmia, dry mouth, and dry eyes, affecting multiple systems. Viral infection can impair eustachian tube ventilation function, causing otitis media; while pharyngeal mucosa exhibits swelling and pain due to viral replication and immune responses (5–7). Chronic viral infections or concurrent bacterial infections can also induce adenoid and tonsil hyperplasia and mucous gland metabolic abnormalities, further weaken mucosal barrier function and increase the risk of secondary infections (8). Children and the elderly are particularly at high risk, often requiring significant medical resources due to severe symptoms and slow recovery, posing severe challenges for clinical prevention and treatment (9). After viral invasion, the host initiates a defense response through innate immunity. Host cells recognize virus-associated molecular patterns (PAMPs) via pattern recognition receptors (PRRs), activating key signaling pathways such as Cyclic GMP-AMP synthase (cGAS)-Stimulator of interferon genes (STING), Retinoic acid-inducible gene I (RIG-I), and Toll-like receptors (TLRs) (10–12). These pathways induce the activation of members of the interferon regulatory factor (IRF) family and nuclear factor-κB (NF-κB), thereby rapidly triggering the expression and secretion of interferons.

Interferons (IFNs) serve as core molecules in the host’s defense against viral infections. Based on structural and receptor differences, they are primarily classified into Type I, Type II, and Type III. Among these, Type I interferons (IFN-I) and Type III interferons (IFN-III) play a critical role in the early stages of innate immunity (13). IFN binds to cell surface receptors to activate the Janus kinase (JAK)–Signal transducer and activator of transcription (STAT) pathway, triggering the expression of numerous interferon-stimulated genes (ISGs). This enables precise inhibition at multiple critical stages of the viral lifecycle, while promoting apoptosis in infected cells and activating effector immune cells such as NK cells, macrophages, and T cells (14–16). In viral infections, IFN-I exhibit strong antiviral efficacy but are prone to inducing systemic inflammation; IFN-III primarily act locally on epithelial cells, causing minimal inflammation, and help inhibit viral spread while maintaining mucosal homeostasis (17, 18). Clinical studies indicate that the timing and intensity of the IFN response directly influence disease progression and prognosis. If the IFN response is insufficient or delayed, viruses may replicate extensively in the early stages, increasing the risk of severe disease; conversely, and excessive activation of IFN signaling may lead to tissue damage or even immune pathologies such as “cytokine storms” (19). Notably, increasing evidence suggests that viruses can interfere with host metabolic pathways to inhibit IFN production and signaling, thereby achieving immune evasion (20).

Recent studies have shown that viral infections are not only a challenge to the immune system but also a profound reprogramming process of the host’s metabolic network. As a parasitic organism, viruses lack cellular structures and rely almost entirely on the host’s metabolic system to complete their life cycle after infecting host cells (21, 22). The viral life cycle typically involves several consecutive steps: initial invasion of host cells, uncoating to release the genome, entry of the genome into the cell nucleus for replication and transcription, synthesis of viral proteins, and final assembly into mature viral particles for release. Non-enveloped viruses typically rely on binding to specific receptors on the host cell surface for direct invasion, while enveloped viruses primarily enter cells through fusion mechanisms between their envelope and the cell membrane. After viral entry, the viral genetic material is transported into the cell nucleus, initiating subsequent stages of the viral life cycle (23–25). Although early viral proteins possess some autonomous regulatory capabilities, viral replication, transcription, and protein translation processes still extensively rely on various host cell macromolecular mechanisms for support. The assembly of new viral particles also requires the participation of these host factors and is ultimately released into the extracellular space via cell lysis or budding. As such, the metabolic activities of host cells play an indispensable key role in viral replication and amplification (26, 27). To meet the virus’s own rapid replication and assembly needs, multiple metabolic pathways in host cells are significantly activated, including carbohydrate, lipid, amino acid, and nucleotide metabolism, forming a dynamic regulatory system intertwining viruses, metabolism, and immunity (28, 29). Meanwhile, viruses can hijack host metabolism to establish an immunosuppressive environment that inhibits IFN production or ISG expression, thereby evading immune clearance (30). Based on this, regulatory strategies targeting metabolic products or metabolic enzymes have become an important direction for novel antiviral interventions, particularly in high-exposure areas such as the upper respiratory tract. Formulations delivering metabolic regulators or metabolic mimetics locally may enhance IFN-dependent immune barriers, providing new theoretical foundations and therapeutic approaches for the prevention and treatment of otolaryngological viral diseases.

## 2 IFNs-mediated innate antiviral immune characteristics

### 2.1 IFN expression

**Figure 1:**
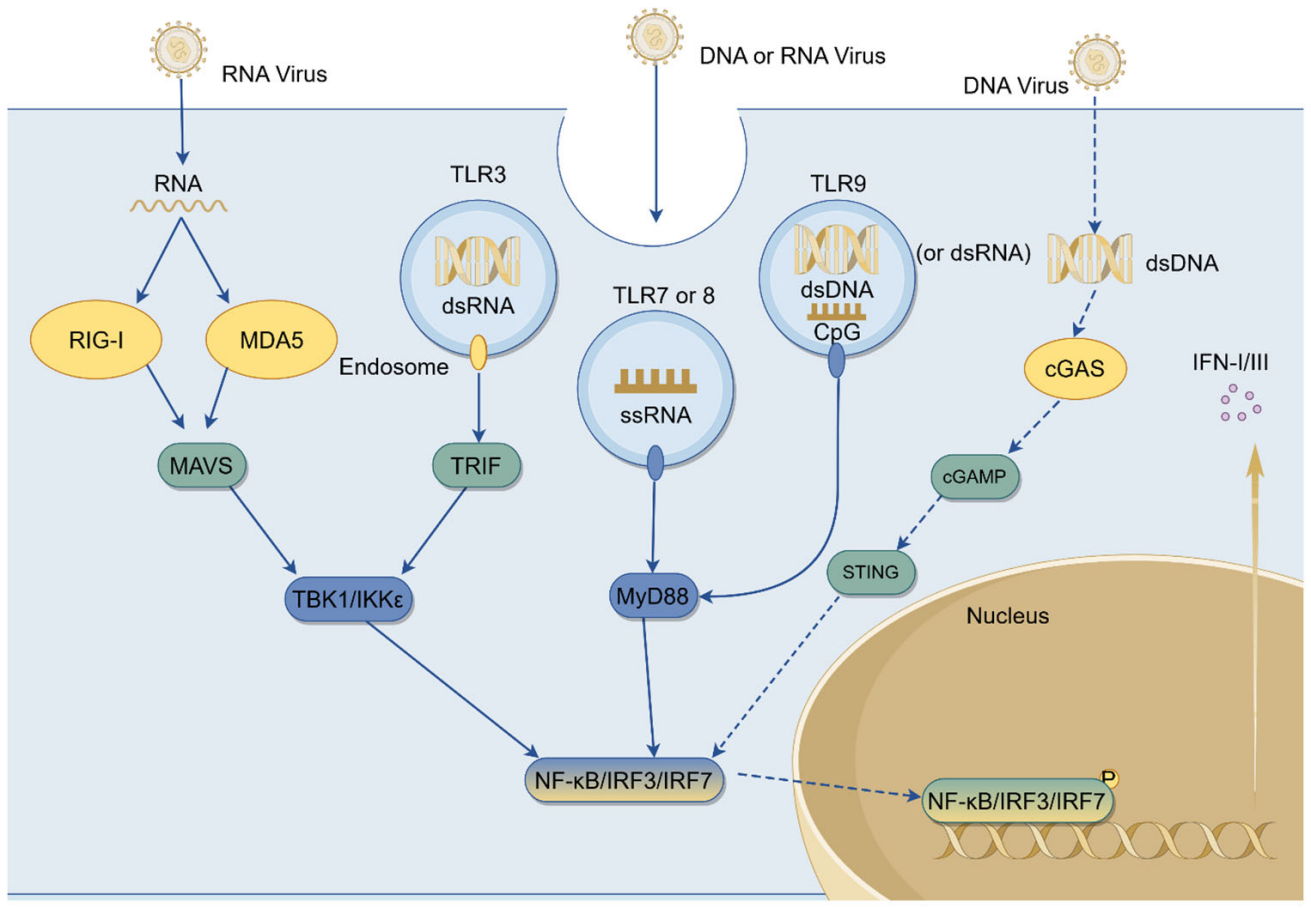
The type I and III interferon signaling pathway. Upon viral infection, RLRs such as RIG-I and MDA5 recognize viral RNA species, including influenza virus, RSV, and HCV. Following activation, RIG-I/MDA5 recruit the adaptor protein MAVS on the mitochondrial outer membrane, which orchestrates the assembly of downstream signaling complexes. This leads to the activation of TBK1/IKKϵ kinases, phosphorylation of IRF3 and IRF7, and activation of NF-κB, all of which translocate into the nucleus to initiate type I and type III interferon transcription and drive the expression of ISGs. In endosomal compartments, TLR3 detects dsRNA from influenza virus or rotavirus through the TRIF pathway, while TLR7/8 recognize ssRNA from viruses such as influenza, dengue virus, and SARS-CoV-2, and TLR9 senses CpG-rich DNA motifs from herpesviruses like HSV and EBV. These TLRs mainly rely on the MyD88 signaling adaptor to activate NF-κB and IRF7, thereby amplifying IFN and pro-inflammatory cytokine production. In the cytoplasm, cGAS detects dsDNA from DNA viruses such as HSV-1, adenovirus, and VZV, catalyzing the synthesis of the second messenger cGAMP. cGAMP in turn activates STING on the endoplasmic reticulum membrane, which recruits TBK1 to phosphorylate IRF3 and stimulate NF-κB, reinforcing IFN production. RLR, RIG-I-like receptor; RIG-I, retinoic acid-inducible gene I; MDA5, melanoma differentiation-associated protein 5; MAVS, mitochondrial antiviral-signaling protein; TBK1, TANK-binding kinase 1; IKK, IκB kinase; IRF, interferon regulatory factor; NF-κB, nuclear factor kappa-B; IFN, interferon; ISG, interferon-stimulated gene; TLR, Toll-like receptor; TRIF, TIR-domain-containing adapter-inducing interferon-β; MyD88, myeloid differentiation primary response protein 88; cGAS, cyclic GMP-AMP synthase; cGAMP, cyclic GMP-AMP; STING, stimulator of interferon genes; HSV, herpes simplex virus; EBV, Epstein–Barr virus; VZV, varicella-zoster virus; RSV, respiratory syncytial virus; HCV, hepatitis C virus.

Otolaryngological regions, including the nasal cavity, pharynx, tonsils, and middle ear, serve as primary entry points for viral invasion due to their anatomical openness and susceptibility of the mucosal barrier. These areas are preferred initial infection sites for various respiratory viruses, including influenza virus, respiratory syncytial virus, rhinovirus, and novel coronavirus. After viral invasion of ENT mucosal epithelial cells, intracellular PRRs recognize pathogen-associated molecular patterns (PAMPs) such as viral nucleic acids and activate key transcription factors such as Interferon regulatory factor (IRF)3/7 and NF-κB through adaptor proteins, thereby initiating the expression of type I and type III IFNs (31, 32). In this process, IFN-I are mainly produced by plasmacytoid dendritic cells (pDCs), monocytes, macrophages, B cells, and certain epithelial cells. Among these, pDCs are considered the most potent source of IFN-I due to their ability to rapidly and abundantly secrete IFN-α (33, 34). Type III interferons, on the other hand, are primarily derived from mucosal epithelial cells and pDCs, playing a dominant role in maintaining local antiviral immunity at epithelial barriers. Collectively, IFN-I and IFN-III exhibit complementary characteristics in terms of tissue distribution and function (35–37). IFN production primarily depends on three classical innate immune signaling pathways: the RIG-I-like receptor (RLR) pathway, the Toll-like receptor pathway, and the cGAS-STING pathway (**Figure 1**). When RNA viruses such as influenza virus, respiratory syncytial virus (RSV), and hepatitis C virus (HCV) infect host cells, cytoplasmic RIG-I and melanoma differentiation–associated protein 5 (MDA5) recognize viral RNA or RNA transcribed from viral DNA, thereby activating the mitochondrial adaptor protein MAVS (38, 39). This in turn activates transcription factors such as IRF3/7 and NF-κB, thereby inducing IFN expression (12). TLRs are primarily located in endosomal compartments, where they detect viral nucleic acids. Specifically, TLR3 recognizes double-stranded RNA (dsRNA) from viruses such as influenza virus and rotavirus (40), TLR7/8 recognize single-stranded RNA (ssRNA) from viruses including influenza virus, dengue virus, and SARS-CoV-2 (41), and TLR9 senses CpG-rich DNA motifs from herpesviruses such as HSV (42) and EBV (43). These TLRs signal through the adaptor proteins Myeloid differentiation primary response 88 (MyD88) or TIR-domain-containing adapter-inducing interferon-β (TRIF), ultimately leading to activation of transcription factors IRF3/7 and the induction of type I interferon responses (44). Cytoplasmic DNA from DNA viruses such as HSV-1, adenovirus, and varicella-zoster virus (VZV) can be recognized by cGAS (45, 46), which catalyzes the production of the second messenger cyclic GMP–AMP (cGAMP). cGAMP subsequently activates the adaptor protein STING on the endoplasmic reticulum membrane, leading to the recruitment of TANK-binding kinase 1 (TBK1), phosphorylation of IRF3, and activation of NF-κB, thereby inducing type I interferon expression (47).

### 2.2 IFN activation of antiviral gene expression

IFN-I bind to the interferon alpha receptor 1/2 (IFNAR1/2) complex on the cell surface, while IFN-III bind to the interferon lambda receptor 1 (IFNLR1)/IL-10Rβ receptor. Both activate receptor-associated Janus kinases, which in turn phosphorylate the signal transducers Signal transducer and activator of transcription (STAT)1 and STAT2. Phosphorlated STAT1/2 form the interferon-stimulated gene factor 3 (ISGF3) complex with IRF9, translocate into the nucleus, and bind to the interferon-stimulated response element (ISRE), inducing the expression of a series of ISGs and initiating a broad-spectrum antiviral program (48, 49). The effector molecules encoded by ISGs, such as MxA, 2’,5’-oligoadenylate synthetase (OAS), and protein kinase R (PKR), can synergistically inhibit viral replication, transcription, translation, and budding in the otolaryngological mucosa through multiple targets, effectively blocking the progression of infection (50–52).

### 2.3 Type I and Type III IFNs

In the local mucosa of the otolaryngology region, Type I and Type III IFNs exhibit complementary and coordinated characteristics in terms of distribution and function. IFN-I rapidly activate a strong systemic antiviral response through the IFNAR receptors widely distributed on the surfaces of immune cells and various structural cells. However, they also tend to induce excessive inflammation, increasing the risk of mucosal tissue damage (53). In contrast, Type III interferon, whose signaling is mediated by the IL-10Rβ receptor confined to mucosal epithelial cells, exhibits more localized and targeted antiviral effects with fewer side effects, making it more suitable for complex mucosal barriers prone to inflammation, such as those in the otolaryngology region (54). Functionally, IFN-I induces rapid and intense ISG expression, but it is short-lived and susceptible to inhibition by negative regulatory molecules such as ubiquitin-specific peptidase 18 (USP18) and suppressor of cytokine signaling 1 (SOCS1). On the other hand, IFN-III induces ISG expression that is longer-lasting, less intense, but more stable, and is less susceptible to interference from the aforementioned negative feedback mechanisms (17). Studies have shown that IFN-III can effectively activate the expression of defense genes in nasal and middle ear mucosa, limit viral replication, while maintaining the integrity of the epithelial barrier and reducing the risk of secondary bacterial infections, thereby demonstrating its unique advantages in mucosal immune defense (55). At the cellular level, ciliated epithelial cells and secretory cells (including goblet and club cells) are the predominant producers of type I and type III interferons during viral infection (32, 56). The basal cells remain highly responsive to both IFN-I and IFN-III, thereby supporting epithelial repair and sustained antiviral protection (57, 58).

## 3 Metabolic reprogramming and antiviral immunity: the key role of metabolites

Cellular pathways of glucose, lipid, amino acid, and nucleotide metabolism occupy a central position in life activities, not only providing energy and biosynthetic precursors for the organism but also actively participating in immune regulation through a variety of intermediate metabolites. Viral infections often induce profound remodeling of the host metabolic network, leading to the reprogramming of critical metabolic pathways. On the one hand, such metabolic restructuring supplies energy and raw materials for viral replication; on the other hand, the resulting metabolic intermediates play pivotal regulatory roles in interferon-mediated innate immune responses, thereby constituting a crucial metabolic interface in the interaction between virus and host (59).

### 3.1 Glucose metabolism

Glucose metabolism represents a fundamental process for sustaining life and constitutes the primary pathway by which cells acquire energy. Through glycolysis, the tricarboxylic acid (TCA) cycle, and oxidative phosphorylation, glucose is catabolized into carbon dioxide and water, generating large amounts of ATP to support essential biological activities such as ion transport, protein synthesis, muscle contraction, and neural transmission. Notably, tissues such as the brain and red blood cells rely almost exclusively on glucose for energy supply (60). Intermediate products generated during glucose metabolism, including pyruvate, lactate, and glucose-6-phosphate, not only provide precursors for the biosynthesis of fatty acids, amino acids, and nucleotides but also fuel the pentose phosphate pathway and glycogen synthesis, thereby supplying ribose-5-phosphate for nucleotide production and maintaining redox homeostasis through NADPH generation (61–63). Moreover, glucose metabolism is intimately linked to immune regulation. Activated macrophages, T cells, and NK cells commonly exhibit upregulated glycolysis to meet the heightened energy and metabolic demands required for rapid proliferation and effector functions. In particular, during the transition of T cells from a resting to an activated state, cellular metabolism shifts from oxidative phosphorylation to a high reliance on glycolysis, maintaining elevated glycolytic flux even under aerobic conditions to rapidly generate ATP and biosynthetic precursors (64, 65).

There exists a broad and profound interaction between glucose metabolism and the antiviral innate immune system. Glucose metabolism not only provides energy and biosynthetic precursors for antiviral immunity but also represents an important target of viral infection–induced reprogramming. Upon infection of the lower respiratory tract with H1N1, local glucose metabolism is significantly enhanced, a response derived from the rapid activation of immune cells (such as macrophages and alveolar epithelial cells) against viral invasion. The underlying mechanism involves stabilization of hypoxia-inducible factor 1α (HIF-1α) and upregulation of key glycolytic enzymes including hexokinase 2 (HK2), pyruvate kinase M2 (PKM2), and pyruvate dehydrogenase kinase (PDK), thereby enhancing “aerobic glycolysis” activity. Even under oxygen-sufficient conditions, cells tend to rely on glycolysis for energy, a phenomenon known as the “Warburg effect.” This metabolic pattern not only accelerates ATP production but also results in the accumulation of intermediates required for the synthesis of nucleic acids, amino acids, and lipids, thereby meeting the metabolic demands of both viral replication and host cell proliferation (66, 67). It is noteworthy that alveolar macrophages (AMs) exhibit distinct metabolic characteristics compared with peritoneal or splenic macrophages. Located in the alveolar microenvironment, which is oxygen-rich, low in glucose, and abundant in surfactant, AMs rely more on oxidative phosphorylation (OXPHOS) and lipid metabolism to maintain homeostasis (68, 69). In low-glucose environments, AMs depend less on glycolysis and instead utilize OXPHOS to fulfill their energy requirements, in sharp contrast to other macrophage populations such as bone marrow–derived macrophages (70, 71). This metabolic adaptability enables AMs to effectively perform immune functions in the highly oxygenated pulmonary environment while preserving homeostasis during inflammatory responses.

A similar phenomenon is observed in Epstein–Barr virus (EBV)–latently infected nasopharyngeal carcinoma (NPC) cells, where latent membrane protein 1 (LMP1) and latent membrane protein 2 (LMP2) cooperatively activate the mammalian target of rapamycin complex 1 (mTORC1)–c-Myc signaling axis. This activation promotes glucose uptake and glycolytic capacity, as well as protein translation efficiency, thereby sustaining viral latency and tumorigenesis. In addition, recurrent mutations in the NF-κB and epidermal growth factor receptor/phosphoinositide 3-kinase (ERBB/PI3K) signaling pathways frequently observed in NPC further activate mTOR, establishing a persistent metabolic drive that constitutes a key mechanism underlying EBV-associated oncogenesis (72).

Glucose metabolism–derived intermediates exert multifaceted regulatory effects on IFN responses. At the metabolic level, glycolytic intermediates such as glucose-6-phosphate, 3-phosphoglycerate, and phosphoenolpyruvate modulate the intracellular nicotinamide adenine dinucleotide oxidized/reduced ratio (NAD^+^/NADH), thereby influencing sirtuin (SIRT) activity and the acetylation status of transcription factors including IRF3, IRF7, and NF-κB, ultimately shaping IFN-β promoter activity and ISG expression (73, 74). At the signaling level, lactate accumulation driven by the Warburg effect, as well as hyperglycemia, suppress IFN-I production by disrupting the MAVS/RIG-I complex and blocking IRF3 and NF-κB activation (75–78). Conversely, activation of the hexosamine biosynthetic pathway (HBP) yields uridine diphosphate N-acetylglucosamine (UDP-GlcNAc), which, through O-linked N-acetylglucosamine transferase (OGT)–mediated modification of IRF5, enhances IFN-I signaling (79). At the epigenetic level, pyruvate-derived acetyl-coenzyme A (acetyl-CoA) not only promotes IFN-I production through reactive oxygen species (ROS) generation (80–82), but also drives histone acetylation, thereby increasing chromatin accessibility and antiviral gene transcription (83). The tricarboxylic acid (TCA) intermediate succinate further acts as a double-edged regulator: it augments inflammation and T cell effector function (e.g., IFN-γ and TNF-α expression), while restraining the MAVS–TBK1–IRF3 pathway to prevent excessive IFN activation and tissue injury (84, 85). Collectively, glucose metabolites fine-tune IFN responses across metabolic, signaling, and epigenetic layers, forming a critical metabolic–immune interface in virus–host interactions.

### 3.2 Lipid metabolism

Lipid metabolism plays a central role in maintaining cellular energy homeostasis, membrane integrity, and signal transduction. Fatty acids, as the most energy-dense form of storage, are deposited as triglycerides in adipose tissue under conditions of nutrient excess. During metabolic stress, they are mobilized through lipolysis and undergo β-oxidation to generate acetyl-CoA, which enters the TCA cycle to produce ATP, thereby ensuring long-term and efficient energy supply (86, 87). Structurally, phospholipids, cholesterol, and sphingolipids constitute the backbone of the bilayer membrane, maintaining membrane fluidity and permeability while regulating the distribution and function of receptors, transporters, and immune recognition molecules via microdomains such as lipid rafts (88). In terms of signaling regulation, lipid-derived metabolites—including arachidonic acid derivatives (prostaglandins, leukotrienes), lysophosphatidic acid (LPA), and sphingosine-1-phosphate (S1P)—act as signaling molecules that precisely regulate cellular proliferation, apoptosis, inflammation, and chemotaxis through G protein–coupled receptors or nuclear receptors (89). In addition, lipid metabolism mediates the absorption and activation of fat-soluble vitamins and contributes to the biosynthesis of steroid hormones, bile acids, and vitamin D, thereby regulating endocrine function and calcium–phosphate metabolism (90, 91). Notably, immune cells such as macrophages and dendritic cells undergo lipid metabolic reprogramming upon activation, which influences antigen presentation, cytokine secretion, and T cell responses, thereby playing a pivotal role in maintaining the balance between immune activation and tolerance (92, 93).

Viral infections are frequently accompanied by remodeling of host lipid metabolism. This metabolic reprogramming not only provides membrane components and energetic support for viral entry, replication, assembly, and budding but also profoundly shapes immune responses, thereby forming a critical metabolic hub in virus–host interactions (94, 95). For example, hepatitis C virus (HCV) downregulates the antiviral activity of miR-130b and miR-185 in interferon-stimulated macrophages and dendritic cells, thereby promoting lipid accumulation and weakening the antiviral effects of the oxysterol 25-hydroxycholesterol (25-HC) secreted by these cells (96). Dengue virus infection drives macrophages toward a metabolic reprogramming characterized by amino acid consumption and enhanced fatty acid and carbohydrate synthesis, which simultaneously supports viral replication and promotes immune activation (97). In respiratory viruses, influenza virus facilitates viral assembly and budding by modulating host cholesterol metabolism and lipid droplet formation; accordingly, inhibition of cholesterol biosynthesis or disruption of lipid droplet biogenesis effectively restricts influenza virus replication (98–100). Similarly, SARS-CoV-2 induces extensive remodeling of host lipid metabolism, with its replication being highly dependent on cholesterol and lipid biosynthetic pathways. Inhibitors of cholesterol metabolism or lipid synthesis blockade have been shown to significantly reduce SARS-CoV-2 replication efficiency (101–103).

**Figure 2:**
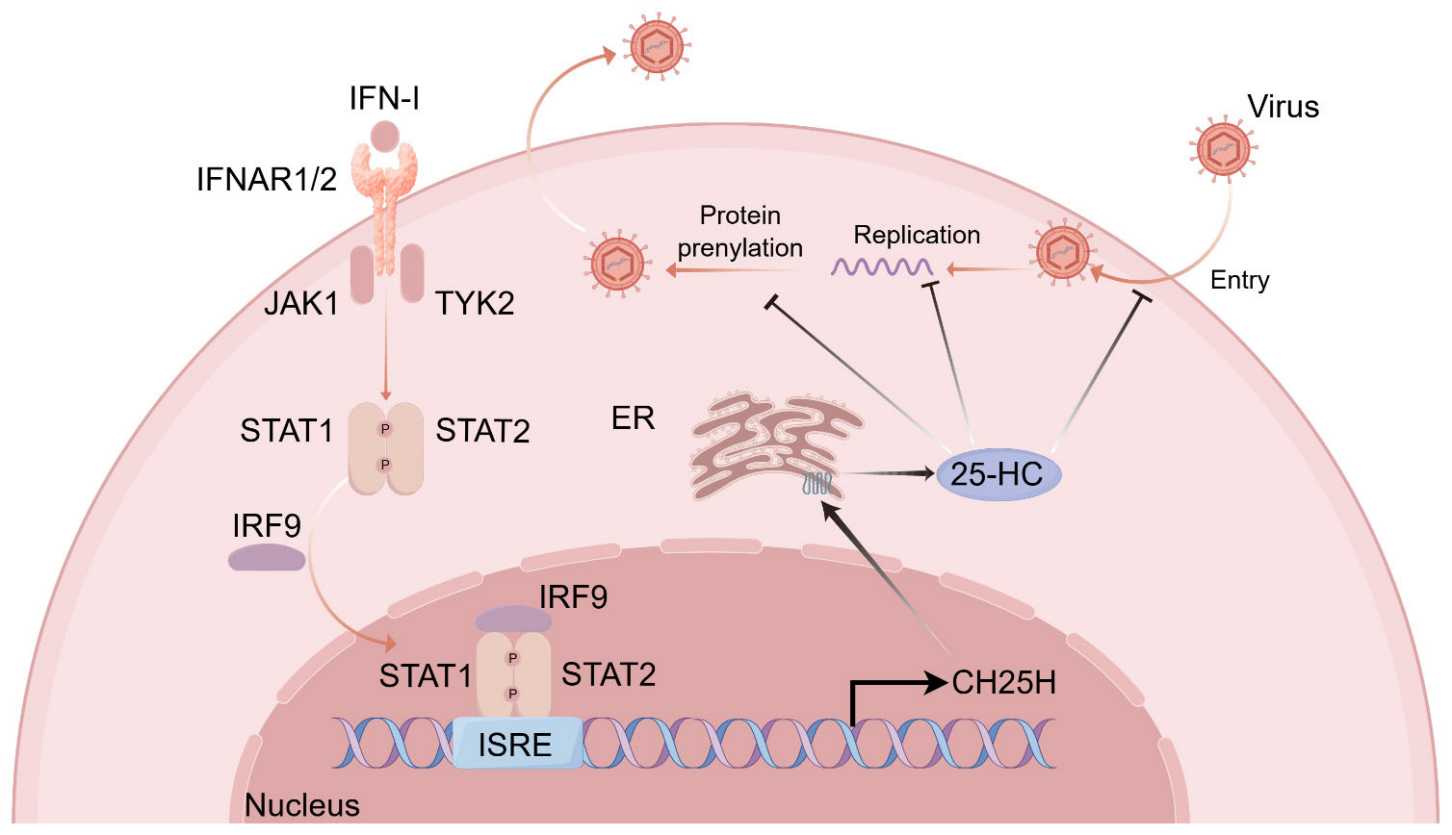
The type I interferon induces 25-HC expression to exert antiviral effects. Upon binding of IFN-I to their receptor complex IFNAR1/2, the receptor-associated tyrosine kinases JAK1 and TYK2 are activated, leading to the phosphorylation of the downstream transcription factors STAT1 and STAT2. The phosphorylated STAT1/STAT2 heterodimer subsequently associates with IRF9 to form the heterotrimeric complex ISGF3, which translocates into the nucleus and binds to ISREs, thereby initiating the transcription of a broad array of ISGs. Among these, CH25H, a prototypical ISG located in the ER, catalyzes the conversion of cholesterol into 25-HC. 25-HC establishes a multilayered antiviral barrier through multiple mechanisms: it lowers the cholesterol content of cellular membranes to inhibit viral attachment and entry; it suppresses viral genome replication; and it antagonizes protein prenylation of both viral and host factors involved in viral replication and assembly, while altering intracellular cholesterol distribution in the ER to further impede viral assembly and packaging. IFN-I, type I interferon; IFNAR1/2, interferon-α receptor subunit 1/2; JAK1, Janus kinase 1; TYK2, tyrosine kinase 2; STAT1/STAT2, signal transducer and activator of transcription 1/2; IRF9, interferon regulatory factor 9; ISGF3, interferon-stimulated gene factor 3; ISRE, interferon-stimulated response element; ISG, interferon-stimulated gene; CH25H, cholesterol-25-hydroxylase; 25-HC, 25-hydroxycholesterol; ER, endoplasmic reticulum.

The host is likewise capable of utilizing specific lipid metabolites to establish antiviral metabolic barriers and modulate immune responses. Among these, 25-HC functions as a key interferon-stimulated effector molecule with central roles in antiviral defense (104). 25-HC is generated by cholesterol 25-hydroxylase (CH25H), a prototypical ISG. Under stimulation by TLR ligands or IFN-I, macrophages and dendritic cells markedly upregulate CH25H expression (105). Mechanistically, 25-HC restricts viral infection through multiple layers: on the one hand, by reducing cellular membrane cholesterol, it blocks virus–host membrane fusion and inhibits viral attachment and entry; on the other hand, by modulating lipid raft structures, it enhances the interactions of MAVS and TRAF with PRRs, thereby amplifying interferon signaling (106). In addition, 25-HC impedes viral genome replication, suppresses host protein prenylation required for viral life cycles, disrupts intracellular cholesterol distribution, and activates the ISR, collectively forming multilayered metabolic barriers against viral replication (104, 107–109). Through its broad involvement in innate immunity, adaptive immunity, and inflammatory regulation, 25-HC provides essential metabolic support for the antiviral effects of interferons (**Figure 2**).

Beyond 25-HC, other lipid metabolites also exhibit significant immunomodulatory potential. Short-chain fatty acids (SCFAs), such as propionate and butyrate, can attenuate inflammatory responses by inhibiting the NF-κB pathway, IFN-β-STAT1 signaling, and histone deacetylase (HDAC) activity, suggesting a potential negative regulatory role during viral infection (110). Alterations in lipid metabolism can further influence interferon production through modulation of fatty acid oxidation, mitochondrial function, ROS levels, and the opening state of the mitochondrial permeability transition pore (mPTP) (111). Notably, certain lipid-derived metabolites possess unique noncanonical antiviral mechanisms. For example, protectin D1 isomer (PDX), derived from omega-3 polyunsaturated fatty acids, has been shown in influenza virus infection models to effectively restrict viral replication by inhibiting viral mRNA nuclear export rather than through conventional inflammatory pathways, highlighting its potential as a promising metabolic intervention strategy (112).

### 3.3 Amino acid and nucleotide metabolism

Amino acid and nucleotide metabolism play central roles in sustaining cellular life processes, serving not only as fundamental precursors for protein and nucleic acid synthesis but also as critical contributors to energy supply, signal regulation, and immune responses. During conditions of energy deficiency or rapid cell proliferation, amino acids can serve as alternative carbon sources entering the TCA cycle to maintain metabolic homeostasis. Among them, glutamine can be converted into glutamate and subsequently into α-ketoglutarate, functioning as an essential substrate for both T cells and tumor cells; alanine and branched-chain amino acids also participate in energy metabolism and the regulation of glucose homeostasis through specific pathways (113, 114). In addition, amino acids also serve as precursors for multiple essential molecules. For example, glycine, aspartate, and glutamine are indispensable for purine and pyrimidine biosynthesis, while cysteine, glutamate, and glycine contribute to glutathione synthesis to maintain redox homeostasis (115, 116). At the immune level, arginine is metabolized by nitric oxide synthase (NOS) to generate nitric oxide (NO), which promotes macrophage activation and pathogen clearance. Tryptophan, on the other hand, is catabolized via the indoleamine 2,3-dioxygenase (IDO)–mediated kynurenine pathway to regulate T cell suppression and regulatory T cell (Treg) induction, thereby playing a critical role in chronic inflammation and immune tolerance (117, 118).

Nucleotide metabolism likewise represents a central hub of cellular life activities. Nucleoside triphosphates such as ATP and GTP serve as the primary energy currency, cyclic nucleotides including cyclic adenosine monophosphate (cAMP) and cyclic guanosine monophosphate (cGMP) function as second messengers, and cofactors such as nicotinamide aden\ine dinucleotide (NAD^+^), nicotinamide adenine dinucleotide phosphate (NADP^+^), and flavin adenine dinucleotide (FAD) drive diverse redox reactions (119). Nucleotides can be obtained through either *de novo* biosynthesis or salvage pathways to meet the distinct requirements of rapidly proliferating cells, such as immune cells and tumor cells (120). More importantly, nucleotide metabolism is deeply embedded in the regulation of innate immunity: fluctuations in intracellular deoxynucleoside triphosphate (dNTP) levels can activate the cGAS–STING pathway to induce IFN-I production, constituting a critical step in viral recognition and antiviral defense (121). At the same time, many viruses hijack host nucleotide biosynthesis to support their replication, whereas the host can establish a “metabolic barrier” by restricting dNTP production to suppress viral propagation (122).

Viral infection often profoundly remodels host amino acid metabolism to meet the demands of protein synthesis and energy supply, while also exploiting metabolic intermediates to regulate immune responses and achieve immune evasion. Vaccinia virus (VACV), for instance, shows a strong dependence on asparagine availability; under glutamine-depleted conditions, exogenous supplementation with asparagine markedly restores viral replication, highlighting the essential role of this amino acid in viral protein synthesis (123). In addition, glutamine itself serves not only as a critical energy source in immune cells such as T cells and macrophages but also enhances IFN–mediated antiviral responses by modulating the expression of ISGs (107, 124, 125). Interferon signaling can actively reprogram metabolic pathways by inducing the expression of specific enzymes. Indoleamine 2,3-dioxygenase 1 (IDO1), a prototypical interferon-stimulated gene, is highly upregulated by both type I and type II interferons. IDO1 catalyzes tryptophan degradation through the kynurenine pathway, leading to tryptophan depletion and restricting the availability of substrates required for viral replication. Kynurenine in turn activates the aryl hydrocarbon receptor (AhR), which promotes the production of immunosuppressive factors such as interleukin-10 (IL-10) and transforming growth factor-β (TGF-β), thereby limiting excessive inflammatory responses. However, sustained AhR activation may suppress interferon signaling, paradoxically favoring viral persistence (112, 126). Another important pathway is arginine metabolism, in which nitric oxide (NO), generated by nitric oxide synthase (NOS), not only directly inhibits viral replication but also synergistically enhances type I interferon signaling, thereby strengthening host antiviral defense (127, 128). Notably, in respiratory viral infections, influenza virus has been shown to substantially alter host glutamine and tryptophan metabolic pathways, driving immune cell functional reprogramming and shaping inflammatory responses—a process closely linked to both antiviral immunity and tissue damage (129).

Nucleotide metabolism likewise occupies a central position in virus–host interactions. Viral replication markedly increases the demand for purine and pyrimidine nucleotides, leading to extensive remodeling of host nucleotide metabolism. For example, dengue virus infection has been shown to broadly affect purine/pyrimidine metabolism, lipolysis, and fatty acid β-oxidation pathways (130). The host, in turn, exploits nucleotide-derived signaling components to activate antiviral responses: cGAS catalyzes the production of the cGAMP, which activates the stimulator of STING–TANK-TBK1–IRF3 signaling axis to strongly induce IFN-I production, thereby providing effective defense against DNA virus infection (131). In addition, energy metabolites such as ketone bodies also possess immunomodulatory functions, capable of influencing interferon responses and epigenetic states, and may hold therapeutic potential in respiratory viral infections (132, 133). SARS-CoV-2 has been demonstrated to hijack host pyrimidine biosynthetic pathways to sustain its replication. Pharmacological blockade of nucleotide biosynthesis—for example, with dihydroorotate dehydrogenase (DHODH) inhibitors—significantly reduces viral replication efficiency, underscoring nucleotide metabolism as a critical metabolic dependency of this virus (134, 135).

Interferonopathies are a group of rare monogenic disorders characterized by chronic overproduction or dysregulation of IFN-I. Although the term “type I interferonopathies” only entered the medical lexicon in 2011, the notion that interferons could be harmful to humans was proposed nearly three decades earlier (136, 137). Metabolic reprogramming may contribute to the pathogenesis of interferonopathies, as sustained IFN-I signaling alters glucose, lipid, and nucleotide metabolism in both immune and parenchymal cells. Indeed, most of the genes reported to be associated with type I interferonopathies are involved in nucleic acid sensing or metabolic pathways (137–139). Thus, incorporating insights from interferonopathies enhances our understanding of the delicate balance between antiviral defense and immunopathology and may open new therapeutic avenues for patients with these conditions through metabolic modulation (**Table 1**).

**Table 1:** Regulatory role of metabolites in IFN signaling.

Metabolic pathways	Key metabolic products	Effects on interferon	Action mechanism	Virus/Bacteria
Glucose metabolism	Lactic acid	Inhibits type I IFN production	Inhibits MAVS/RIG-I complex aggregation; interferes with IRF3/NF-κB activation (75–77).	IAV (75–77)
	Glucose-6-phosphate and other intermediates	Bidirectionally regulates IFN signaling	Regulating the NAD^+^/NADH ratio affects SIRT activity, thereby regulating the acetylation status of IRF3, IRF7, and NF-κB, and modulating the transcriptional activity of IFN-β (73, 74).	RuV (74)
	UDP-GlcNAc	Promotes IFN-I production	OGT-mediated O-GlcNAc glycosylation enhances IRF5 stability, thereby promoting the expression of IFN-I and ISGs (79).	IAV (79)
	Acetyl coenzyme A	Promotes IFN signaling	By promoting histone acetylation, IFN gene chromatin is kept in an open state, while regulating HATs/HDACs activity to enhance transcription factor function and inhibit lipid synthesis, thereby limiting viral replication and assembly (80–83).	VSV (81) IAV (82)
	Succinic acid	Inhibits IFN production	Inhibiting the MAVS-TBK1-IRF3 pathway weakens the type I interferon response and affects IFN-γ secretion by regulating T cell activity (84) (85).	VSV (85)
Lipid metabolism	25-hydroxy cholesterol (25-HC)	Promotes IFN antiviral effects	Lowering membrane cholesterol content blocks viral fusion, enhances MAVS/TRAF signaling to amplify the interferon response, while inhibiting viral replicase activity and activating the ISR (104, 107, 108).	reovirus (107) VSV, HSV, HIV, MHV68, EBOV, RVFV, RSSEV, Nipah viruses (108)
	Short-chain fatty acids (SCFAs)	Inhibits IFN inflammatory responses	Inhibiting NF-κB/STAT1 signaling and HDAC activity alleviates inflammatory responses and may exert a negative regulatory effect on antiviral immunity (110).	Staphylococcus aureus (110)
	Protectin D1 (PDX)	Non-classical antiviral	Inhibiting viral mRNA nuclear export directly limits viral replication (112).	IAV (112)
Amino acid metabolism	Canine urea	Inhibits IFN inflammatory response	Activates the aromatic hydrocarbon receptor (AhR), promotes IL-10 and TGF-β expression, and inhibits IFN-β and IFN-γ (112, 126).	IAV (112, 126)
	Nitric oxide (NO)	Enhances IFN effect	Directly inhibits viral replication and synergistically enhances interferon pathway activation (127, 128, 140).	COVID-19 (140)
	Glutamine	Promotes IFN response	Supports energy supply to immune cells and enhances ISG expression (107, 124, 125).	reovirus (107)
Nucleotide metabolism	cGAMP	Strongly promotes type I IFN production	Activation of the cGAS-STING-TBK1-IRF3 pathway drives type I interferon synthesis (131, 141).	HSV-1 (141)
Pyrimidine biosynthesis	UMP and downstream nucleotidesUMP	Inhibition of IFN-I responses	DHODH inhibitors block nucleotide supply, suppress replication, and may enhance IFN-mediated antiviral effects (134, 135).	COVID-19 (134, 135)

In summary, amino acid and nucleotide metabolism establish intimate connections between viral infection and interferon-mediated immune responses. They not only provide the essential substrates for viral replication but also serve as key nodes for host regulation of interferon production and antiviral effector functions. These metabolic “interfaces” are emerging as important targets in antiviral research, offering new directions and theoretical foundations for the development of metabolism-based antiviral strategies in the future.

## 4 Summary and outlook

Viral infections, especially upper respiratory tract viral infections, have become a major challenge in global clinical prevention and treatment due to the vulnerability and susceptibility of mucosal barriers. IFN-mediated innate immune responses are the core mechanism by which hosts defend against viral infections. However, viruses evade the immune system by reshaping the host’s metabolic environment, thereby increasing the difficulty of clinical prevention and treatment. To address this issue, this paper provides a detailed account of the metabolic reprogramming of host cells during viral infection, focusing on the precise regulatory mechanisms of key metabolic products (such as lactate, 25-hydroxy cholesterol, canine uric acid, and cGAMP) on the interferon pathway. This study reveals that virus-induced metabolic changes affect IFN expression and activity and determine the pathological outcome of infection. In the future, by identifying the key interaction points between metabolic products and IFN, it may be possible to develop novel therapeutic drugs centered on metabolic regulation. Combining these with existing IFN therapies to form localized precision delivery strategies could enhance the efficacy and safety of antiviral treatments, ultimately improving clinical outcomes for otolaryngological diseases.
